# Hypertension and Its Impact on Stroke Recovery: From a Vascular to a Parenchymal Overview

**DOI:** 10.1155/2019/6843895

**Published:** 2019-10-14

**Authors:** Benjamin Maïer, Nathalie Kubis

**Affiliations:** ^1^INSERM U965, CART, Paris, France; ^2^INSERM U1148, Laboratory for Vascular Translational Science, Paris, France; ^3^Sorbonne Paris Cité, Université Paris Diderot, Paris, France; ^4^Service de Physiologie Clinique-Explorations Fonctionnelles, APHP, Hôpital Lariboisière, Paris, France

## Abstract

Hypertension is the first modifiable vascular risk factor accounting for 10.4 million deaths worldwide; it is strongly and independently associated with the risk of stroke and is related to worse prognosis. In addition, hypertension seems to be a key player in the implementation of vascular cognitive impairment. Long-term hypertension, complicated or not by the occurrence of ischemic stroke, is often reviewed on its vascular side, and parenchymal consequences are put aside. Here, we sought to review the impact of isolated hypertension or hypertension associated to stroke on brain atrophy, neuron connectivity and neurogenesis, and phenotype modification of microglia and astrocytes. Finally, we discuss the impact of antihypertensive therapies on cell responses to hypertension and functional recovery. This attractive topic remains a focus of continued investigation and stresses the relevance of including this vascular risk factor in preclinical investigations of stroke outcome.

## 1. Introduction

Hypertension is the first modifiable risk factor, accounting for 10.4 million deaths and 218 million attributable disability-adjusted life-years worldwide [1]. With a recently revised definition (a threshold shift from 140/90 mmHg to 130/80 mmHg), hypertension may concern nearly 50% of the US population [2, 3] and thus is a truly crucial public health issue, especially as hypertension is still dramatically underdiagnosed. In a recent international study population that aimed to estimate hypertension prevalence, awareness, and medication control, only 46.5% of participants were aware that they suffered from hypertension, and only 32.5% of treated patients were pharmacologically controlled [4]. In line with this evidence, the French cross-sectional study ESTEBAN assessed between 2014 and 2016 the same 50% proportion of hypertension awareness and that only 47.3% patients were treated, while again only 55% were pharmacologically controlled [5].

The brain is a major end-organ target of chronic hypertension, leading to an increased risk of stroke and dementia [6–8]. Indeed, hypertension is strongly, independently, and linearly associated with the risk of stroke [6]. At the acute phase of ischemic stroke (IS), hypertension is also extremely frequent [9] and associated with worse functional outcome, mortality, and postthrombolysis symptomatic intracranial hemorrhage [10–13]. Chronic hypertension affects cerebral vessels and functions, notably leading to vascular insufficiency [8]. Consequently, brain parenchyma is also affected. The brain neuroimaging STRIVE (STandards for ReportIng Vascular Changes on NEuroimaging) [14] depicts several isolated or combined hallmarks, such as small subcortical infarcts, lacunes, white matter hyperintensity, perivascular space enlargement, cerebral microbleeds, and brain atrophy, as the most common radiological features associated with chronic hypertension [14], besides large hemorrhagic and ischemic strokes. On the long-term, hypertension has been strongly associated to cognitive decline [15, 16] probably through impaired vasoreactivity and inappropriate neurovascular coupling [17]. Actually, some randomized controlled trials of blood pressure lowering in hypertension have yielded to an improvement of cognitive decline [18–20]. Increased blood pressure at the acute phase of stroke has been reported to increase the probability and severity of poststroke dementia [21], while the impact of chronic hypertension is still a matter of debate [22, 23].

Considering the high worldwide prevalence of hypertension and its deleterious consequences *per se* on brain functions and in the stroke setting, there is an urgent need to fully understand causative mechanisms. Adequate preclinical models, especially including typical comorbidities present in stroke patients, have now been highly recommended for years by the Stroke Therapy Academic Industry Roundtable (STAIR) [24] but still too poorly applied. After briefly addressing rodent hypertension and ischemic stroke (IS) models, we sought to review the impact of isolated hypertension or hypertension associated to IS on brain nonvascular cells and consequences on function. Finally, we discuss the impact of antihypertensive therapies on cell responses to hypertension and functional recovery.

## 2. Rodent Models of Hypertension and Ischemic Stroke

### 2.1. Rodent Models of Hypertension

Several rodent models have been proposed to mimic the hypertension etiologies observed in humans [25–28]. Briefly, they can be categorized into pharmacologically induced hypertension, genetic models, environmental models, and surgically induced models [25] (Table 1). Pharmacologically induced hypertension models are often used in preclinical research since they can be applied either to mice or rats. They consist of an oral or subcutaneous administration of pharmacoactive drugs involved in hypertension (namely angiotensin II (Ag II), deoxycorticosterone acetate (DOCA), and N-nitro-L-arginine methyl ester (L-NAME)), with the duration of treatment depending on the severity of hypertension that is targeted. As it will be addressed below, genetic models are by far the mostly used models for the study of the cerebral consequences of chronic hypertension. Spontaneously hypertensive rats (SHR), originally derived from Wistar Kyoto rats [29], develop hypertension between 2 and 4 months after birth and reach a systolic blood pressure (SBP) peak of 180-200 mmHg by 6 to 7 months and beyond. Close to SHR, stroke-prone SHR (SHRSP), in addition to high blood pressure values, develop earlier and more systematically severe strokes. Consequently, this model has been the cornerstone of works assessing poststroke recovery in hypertensive animals. The Dahl salt-sensitive rats, derived from Sprague-Dawley rats, become hypertensive when a normal salt intake is given [28] and a severe and morbid hypertension with a high salt intake [28]. Other genetic models have been developed [28], among them the Lyon hypertensive rat, the Sabra model, and the Milan SHR. Finally, surgically induced hypertension models have been developed to mimic human renovascular hypertension: the two-kidney one-clip model (i.e., constriction of only one renal artery), the two-kidney two-clip model (i.e., aortic constriction or constriction of both renal arteries), or the one-kidney one-clip model (i.e., constriction of one renal artery and ablation of the contralateral kidney). These techniques will therefore lead to a chronic overactivation of the renin-angiotensin-aldosterone system and a plasmatic increase of the aforementioned molecules, leading to the development of a hypertension that is usually considered as “secondary hypertension” in human practice. The characteristics of these models are also displayed in Table 1.

Unfortunately, each hypertension model recapitulates only one of the numerous pathophysiological pathways that may be involved in human hypertension. Thus, depending on the issue being studied, several hypertension models should be required to ensure that all of these pathways are considered before the translational approach.

### 2.2. Rodent Models of Focal Ischemic Stroke

A large repertoire of rodent ischemic stroke models is required to address the polymorphic facets of this disorder both at the acute and chronic phases to develop poststroke therapeutic strategies. Ideally, an animal stroke model should mimic the successive stages that are encountered in humans, namely, the development of the ischemic core and penumbra [30, 31] and the establishment of a collateral circulation by leptomeningeal anastomoses, the efficacy of which determines the extension of the ultimate lesion [32], but should also induce a sensorimotor deficit at the acute phase and a poststroke cognitive decline at the chronic phase. In that respect, variations between species and strains regarding the vascular structures (i.e., polygon of Willis, extra and intracranial anastomoses, and leptomeningeal collaterals) or immunologic repertoire [33], which will have a major role in neurorepair, must be considered and tailored to the investigation, so that the best model is chosen, and as formulated by Sommer [34]: “best means most closely mimicking a certain aspect of the multiple facets of ischemic stroke”.

The most frequently used rodent ischemic stroke model is the transient or permanent intraluminal middle cerebral artery occlusion (MCAO) using a monofilament with generally large subcortical ± cortical infarcts (Table 2). Transient occlusion, after the withdrawal of the monofilament, leads moreover to a reperfusion according to a preplanned timeframe that closely mimics the ischemia/reperfusion of MCA occlusion treated by mechanical thrombectomy [34–36]. The direct cauterization or suture of a distal branch of the MCA after craniectomy promotes highly reproducible, smaller cortical infarcts, with low morbidity, and it is being brought closer to reality and being developed to study long-term neurorepair processes [37].

Other focal ischemic stroke models in the rodent are displayed in Table 2 and are well reviewed elsewhere [34–36, 38].

## 3. Long-Term Hypertension Has Consequences on Brain Nonvascular Cells

### 3.1. Hypertension-Induced Microglial Polarization Promotes a Proinflammatory State

**Figure 1:**
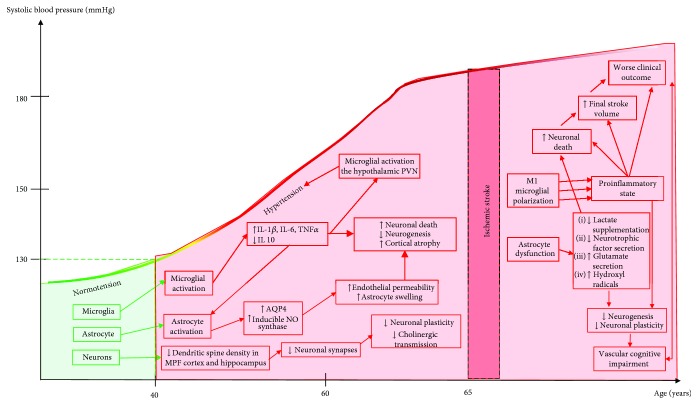
Nonvascular consequences of long-term hypertension and after IS. After IS, hypertension promotes a proinflammatory state by polarizing microglia into a M1-phenotype, thus leading to an astrocyte dysfunction and a decreased neurogenesis and increased neuronal death. Overall, hypertension and IS lead to worse functional outcome and vascular cognitive impairment after IS. On the abscissa level, the age corresponds to the average age of hypertension development and first stroke. MPF: medial prefrontal cortex; AQP4: aquaporine 4; NO: nitric oxide; IL: interleukin; PVN: paraventricular nucleus; SBP: systolic blood pressure.

Adult microglia are derived from erythromyeloid progenitors from the yolk sac and takes up residence early in the neural tube to colonize the future central nervous system (CNS) [39], constituting nearly 10% of the total cells [40, 41]. Under physiological conditions, these cells display numerous fundamental properties and functions such as immunological surveillance [41, 42], synaptic pruning during CNS development [41, 43, 44], synaptic maturation, and synaptic plasticity [41]. Numerous studies have recently brought to light microglia susceptible to hypertension and their possible role in the onset of neurogenic hypertension, driven by the sympathetic nervous system, and its resistant form. Indeed, hypertension induces microglial activation in the hypothalamic paraventricular nucleus (PVN) and induces microglial cells participation in the proinflammatory state with the local secretion of IL-1-beta, IL-6, and TNF-alpha and a decrease of anti-inflammatory cytokines, notably IL-10 [45]. Prohypertensive signals such as angiotensin II activate PVN microglia and increase sympathetic nerve activity through preganglionic sympathetic neuron activation that in turn increases inflammatory cells of the bone marrow, some of them migrating to the PVN and exacerbating thereby neuroinflammation [46]. With the secretion of proinflammatory cytokines, activated microglia also release chemokines and reactive oxygen species (ROS), which sustain the state of “sympathoexcitation” and the perpetuation of hypertension [46]. The participation of the proinflammatory state in the onset of hypertension is also illustrated by the attenuation of hypertension after reducing the inflammation status with intracerebroventricular infusion [45] or oral delivery of minocycline [46] or with the overexpression of IL-10 in the PVN [45]. Taken together, these observations unveiled a previously undescribed impact of hypertension on microglial activation and the impact of neuroinflammation in the onset and maintenance of hypertension [45–47]. In the long term, hypertension maintains a proinflammatory state with increased astrocyte levels and microglial activation, increased plasma levels of proinflammatory cytokines [48], increased leukocyte activation in the spleen [49], and an upregulation of brain endothelial cell adhesion molecules [50–52] (Figure 1).

These data are of utmost importance since they insist on the implementation of a deleterious, highly proinflammatory environment promoted and increased by hypertension, prior to any parenchymal lesion (i.e., stroke). As it will be addressed below, this inflammatory state paves the way for parenchymal damages and might partly explain the limited endogenous potential for recovery in hypertensive stroke patients.

### 3.2. Hypertension-Induced Structural and Functional Alterations of Astrocytes

Astrocytes are the most abundant cell type in the CNS [53] and have key functions regarding the development and homeostasis of the normal brain. Nonexhaustively, these functions include ion buffering and homeostasis [53, 54], neurotransmitter recycling [53], cytokine production [55, 56], blood-brain barrier maintenance and cerebral blood flow regulation [17, 57], neurotrophin secretion [58], synaptic pruning, and immune signaling. During these last years, tremendous efforts have been made to better characterize these cells, in particular their wide heterogeneity depending on the anatomical brain region and cortical layers where they are found, and their responses to pathological conditions [53], such as in neurodegenerative diseases, epilepsy, brain tumors, or stroke. As for microglial cells, a recent study has shed light on the neurotoxic effects of proinflammatory reactive astrocytes A1 after induction by microglia with IL-1-alpha, TNF-alpha, and C1q [59] in different neurodegenerative diseases. Few studies have investigated the effects of long-term hypertension on astrocytes [56]. One of the first descriptions of the ultrastructural changes of astrocytes in SHRSP was performed by Tagami et al. [60] in comparison to age-matched normotensive WKY. Pericytes around the capillaries degenerated during hypertension development, which in turn led to a circumferential astrocyte swelling, opening of interendothelial junctions, increased blood-brain barrier permeability, and neuronal death [60]. Other works have highlighted the more pronounced astrocyte reaction in the setting of hypertension [61] and its impact on endothelial junctions [62]. More recently, the association between hypertension and astrocyte swelling has been illustrated by an overexpression of astrocyte aquaporine-4 (AQP4) in 6-month-old SHR in which hypertension is settled, but not in the prehypertensive phase in 2-month-old SHR, nor in the developing phase of hypertension in 4-month-old SHR in comparison to age-matched WKY [63, 64]. Considering the importance of astrocyte-neuron communication (i.e., the neurovascular unit) [17], astrocyte swelling induced by hypertension could have significant repercussions on neuron physiology and brain plasticity. New studies are needed to assess if neurotrophic factors or proinflammatory cytokine or chemokine secretion by astrocytes are impaired in hypertension; new studies are also needed to assess their potential negative impact.

In SHRSP, reactive astrocytes strongly express inducible NO synthase (iNOS) [65] in comparison to normotensive control rats. The distribution of iNOS+ astrocytes colocalized with the typical brain histological lesions observed in this model, i.e., petechiae and edema. Based on these findings, it was postulated that hypertension led to iNOS expression in reactive astrocytes and that the NO thus generated may be involved in the development of hypertensive cerebral lesions [65]. iNOS is induced by proinflammatory states, particularly by the innate immune system and microglia in particular. Thus, the hypertension-induced microglial polarization towards a proinflammatory state could contribute to the proinflammatory astrocyte polarization (i.e., A1) and enhance neurotoxic effects [59]. These abovementioned inflammatory alterations induced by long-term hypertension must be taken into account to apprehend the neuronal impact of long-term hypertension.

### 3.3. Hypertension Is Responsible for Cortical Atrophy and Impaired Hippocampus Neurogenesis and Leads to Cognitive Impairment

Hypertension causes profound macroscopic and microscopic alterations of the brain parenchyma. Described in the early 1980s, SHR brains present significantly reduced weights and volumes [66, 67], enlarged cerebral ventricles as early as 8 weeks old [68], and cortical atrophy in several cortical areas [68] in comparison to age-matched WKY. Overall, neuronal density is globally reduced in most brain regions [67]. These studies were the first to highlight the neuronal consequences of long-term hypertension but did not specifically assess the brain structure involved in long-term memory (i.e., hippocampus). Similarly, no behavioral and cognitive analyses accompanied these histological studies. This issue was addressed in the early 2000s: microanatomical studies of hippocampus in SHR showed hippocampal atrophy and the aggravating role of age [69, 70]. Indeed, still not significantly different in 2-month-old SHR and WKY rats, the hippocampus volume was reduced by 16% and 21% in, respectively, 4- and 6-month-old SHR compared to WKY. Four-month-old SHR presented a decreased volume of CA1 whereas a grey matter volume reduction in the CA1 and in the dentate gyrus were evidenced in 6-month-old SHR [69]. In these 6-month-old SHR, 5% of the neurons in the CA1 subfield displayed microanatomical characteristics of damaged cells with apoptosis and necrosis [69]. This study was therefore one of the first to address the direct consequences of hypertension in the hippocampus but did not present any pathophysiological explanation regarding the specific mechanism of this atrophy. Later on, signs of neurodegeneration were observed in the hippocampus of 6-month-old SHR, with a reduced level of the 200-kDa cytoskeletal phosphorylated neurofilament [70]. Diffuse atrophy, and in particular hippocampus atrophy secondary to hypertension, could therefore share mechanisms with neurodegenerative diseases, such as Alzheimer's disease. Neurogenesis, in the setting of hypertension, was thus evaluated.

Neurogenesis in the human brain takes place in part in the dentate gyrus of the hippocampus [71]. At first, several lines of evidences surprisingly pointed to a possible increased neurogenesis in hypertension, as demonstrated by double Ki-67 and doublecortin (DCX) immunostaining in 13-week-old SHR and SHRSP rats compared to WKY [72]. Interestingly, a correlative association between blood pressure values and adult hippocampal neurogenesis was observed only in SHRSP rats. This increased neurogenesis in both SHR and SHRSP strains was achieved through an increased cell proliferation rate in SHR animals and an increase survival of newly formed neurons in SHRSP [72]. Sex also matters. A higher number of new neurons in young SHR was observed compared to age-matched Sprague-Dawley rats, but males from both strains generated more cells than female counterparts [73]. By contrast, despite increased neurogenesis in SHR at 1, 8, and 12 months compared to Sprague-Dawley rats, it finally decreased with age in both groups [74]. Dahl salt sensitive (DSS) with high-salt diet and chronic intermittent hypoxia (CIH), which mimics hypertension observed in patients with obstructive sleep apnea, showed that the latter model only induced neurogenesis in the dentate gyrus [75]. By comparing neurogenesis in the dentate gyrus by BrdU staining between young (1-month-old) SHR, salt-sensitive/salt-resistant strains of Dahl rats, and SHR controls treated by captopril, no significant difference was evidenced [76]. In older hypertensive animals such as 16-week-old SHR, WKY, and Sprague-Dawley with and without deoxycortisone acetate (DOCA) and salt, a constant decreased cell proliferation rate was observed in the dentate gyrus [77]. However, one must keep in mind that no clinical evaluation of cognitive impairment was assessed in these studies. In a nongenetic murine renovascular hypertension model (3-month-old male C3H/HeJxC57Bl6 mice subjected to the “two-kidney one-clip” technique), long-term memory assessed by the Morris water maze was impaired in hypertensive mice and hippocampal neurogenesis was reduced in comparison to normotensive mice associated with a reduced level of hippocampal brain-derived neurotrophic factor (BDNF), a major plasticity factor [78]. Thus, hypertension animal models, strains, age, and sex may account for the discrepancy in neurogenesis assessment between studies. However, whether newborn neurons promote an aberrant hippocampal circuitry remodeling has not yet been investigated.

Interestingly, long-term hypertension affects dendritic spine density in crucial brain regions implicated in cognition [79]. Four-month-old SHR display decreased spine density in pyramidal neurons of the medial prefrontal cortex and in medium spiny cells of the nucleus accumbens, in comparison to age-matched WKY [80]. In addition, 8-month-old SHR also exhibit a decrease in the number of dendritic spines in the CA1 pyramidal cells [80], as assessed by the Golgi-Cox impregnation method, a technique that identifies the dendritic surface, an area that receives in physiological conditions more than 95% synapses for a given neuron [80, 81]. In renovascular hypertension, after 16 weeks of sustained hypertension, WKY rats exhibited a 50% decrease in dendritic spine density of prefrontal cortical pyramidal neurons compared to sham age-matched animals [82]. As the number of dendritic spines is related to the degree of connectivity and afferent activity, it has been suggested that long-lasting hypertension impairs neural plasticity by decreasing the number of synapses [80, 83]. Taken together, these results highlight that synaptic communication may be altered in the setting of hypertension [79] (Figure 1).

From a biochemical point of view, Amenta et al. pointed out impaired cholinergic functions in SHRSP in comparison to age-matched WKY [84]. Interestingly, SHRSP exhibited a decreased level of acetylcholine and choline within the cerebral cortex, the hippocampus, and the cerebrospinal fluid [84–87], which was associated with cognitive impairment [84, 88]. This impairment of cholinergic function has also been assessed at the synaptic level in the cerebral cortex and the hippocampus of SHRSP [89] and SHR, respectively, with a decreased number of nicotinic cholinergic receptors and reduced binding sites in comparison to age-matched WKY [90].

Taken together, these studies indicate that hypertension leads to neuronal loss, decreased dendritic spines and cholinergic neurotransmission, impaired neurogenesis, and consequently, altered neural plasticity, partly explaining the cognitive decline exhibited by hypertensive mice [78]. Further studies are needed to exhaustively understand the mechanisms underlying impaired neuronal plasticity and, in particular, the relationship with neuroinflammation.

Hypertension is found in half of the patients suffering from acute IS [12] as a vascular risk factor prior to stroke or complicating the stroke time course at the acute phase. It has been shown to affect clinical outcome by decreasing brain plasticity as depicted in Section 4.

## 4. The Combination of Hypertension to IS Worsens Clinical Prognosis

### 4.1. After Ischemic Stroke, Hypertension Drives Microglial Polarization and Promotes a Deleterious Proinflammatory State

It is now widely accepted that inflammation and immunology are key players not only in the early postischemic period but also in the long term and will impact on long-term recovery [37, 91, 92]. Innate immune cells of the central nervous system, embodied by the microglial cells, seem to be the earliest to be activated [93]. Briefly, immediately after the sudden interruption of cerebral blood flow, dying cells release Danger Associated Molecular Pattern (DAMP) molecules [41], recognized by pattern recognition receptors such as Toll-Like Receptors, highly expressed at the surface of microglia [41, 91, 94]. They induce microglial activation which switches from a ramified resting phenotype to an amoeboid activated phenotype. Schematically, activated microglia may either promote an anti-inflammatory response with the enhancement of phagocytosis thanks to the M2-phenotype (being the extreme form of anti-inflammatory microglia) [95, 96] and the secretion of IL-10 and TGF-beta or a proinflammatory state with the M1-phenotype microglia (being the extreme form of proinflammatory microglia) and the secretion of proinflammatory cytokines, such as IL-1-beta, TNF-alpha, IFN-gamma, and IL-8. This proinflammatory state, if prolonged, is associated with an increased infarct volume, worse sensorimotor scores, systemic infection, decreased neural plasticity, and death [91], justifying the growing interest of scientists regarding the use of immunomodulatory therapies in order to tone down this inflammatory state.

In the setting of IS, hypertension also plays a role in microglial polarization and the establishment of a proinflammatory state associated with increased infarct volume and worsened functional outcome [97, 98]. SHRSP display a significant increase of activated microglia compared to WKY not only in the infarct core and the peri-infarct area but interestingly also in the contralateral hemisphere [98]. Those observations used immunochemistry and were later confirmed by flow cytometry [52, 99], but several discrepancies regarding microglial activation and infiltration of myeloid cells after ischemic stroke remain, according to the stroke animal model, strain, and age studied. In SHR, in which cerebral ischemia was induced by the thermocoagulation of a distal branch of the middle cerebral artery, neutrophils, monocytes, and myeloid dendritic cells entered the brain at day 1, whereas microglial cell counts were comparable with the control condition, but increased by almost 300% at day 4 with a concomitant decrease of neutrophils and monocytes [99]. Unfortunately, no controls (i.e., normotensive animal) were available to compare the leucocyte response. In the second work of the same team, young (12-14 weeks) WKY and SHR rats were subjected to photothrombosis, and the time course of leucocyte infiltration and microglial activation was assessed after stroke [52]. Using flow cytometry at day 4, there was a fourfold increase in CD45+ cells in the SHR ischemic brain in comparison to WKY. These cells were in majority neutrophils, monocytes, and macrophages, which were all increased with a higher rate of neutrophils and monocyte chemokines in SHR (CCL2, CCL3, and CXCL2) in comparison to WKY. In this model, proinflammatory M1 macrophages/microglia and M2 anti-inflammatory macrophages/microglia were in the same proportion in SHR and WKY, so were the classical M1 (IL-1-beta, IL-6, and TNF-alpha) and M2 markers (IL-10, TGF-beta) [52]. However, in an endothelin-1 stroke model, opposite results regarding microglial activation were found. In comparison to age-matched WKY, SHR displayed reduced microglial activation defined by CD68+ and Iba-1 staining 3 days after stroke onset but an increase in final infarct volume [100]. Those results were then confirmed with *in vitro* analysis, assessing microglial responsiveness to lipopolysaccharide (LPS), showing a decreased microglial activation in SHR. The discrepancies between the studies mentioned above might be explained by the different stroke models and by the absence of specificity of the immunohistochemical markers between microglia and macrophages and between the different microglial subpopulations (M0, M1, M2a, M2b, and M2c) [95], highlighting the necessity to combine flow cytometry analysis with different strains and ischemic stroke models in the context of hypertension.

### 4.2. After Ischemic Stroke, Hypertension Worsens Astrocyte Activation and Reduces Its Neuroprotective Effects

Despite lots of evidences that support the colossal involvement of astrocytes in the setting of IS, there is a controversy regarding their exact role and function in IS prognosis [101–104]. This can be explained by the timing of analysis after IS (acute versus long term), their localization inside the ischemic core or in the ischemic penumbra, the stroke model, and the proinflammatory or anti-inflammatory phenotype [53, 59]. Indeed, after stroke, astrocytes [105] upregulate Glial Fibrillary Acidic Protein (GFAP) and Vimentin genes, leading to morphological changes to astrocyte activation [106–108] and the formation, within the ischemic penumbra, of a glial scar that is associated with astrocytes, microglia/macrophages, and extracellular matrix molecules, chondroitin sulfate proteoglycan in particular [109, 110]. The exact function of this glial scar has been extremely debated with studies finding a beneficial effect (limitation of leukocyte infiltration [108, 111], central nervous system axonal regeneration [109]) or, on the contrary, limited neurite outgrowth [112, 113], mostly depending on the analysis timeframe. On the long term, recent studies point to the fact that reactive astrocytes could have a deleterious impact on poststroke recovery through inhibiting synaptic plasticity [108, 114]. Those so-called contradictory results reflect the huge heterogeneity of astrocytes and functions according to different regional chemical conditions. In *in vitro* hypoxia/reoxygenation (H/R) models, the lactate astrocyte production was weaker in SHR in comparison to WKY, leading to a neuronal energy deficiency and neuronal death [56, 115]. This observation was partly explained by a downregulation of transmembrane lactate transporters such as monocarboxylate transporter 1 (MCT-1) [115]. Despite the debated effect of cerebral lactate in the setting of IS [116], many evidences point to the beneficial effect of astrocyte lactate production on neuronal survival, specifically within the ischemic penumbra. Indeed, most glycogen stores in the adult brain being found in the astrocytes [103, 117], the conversion of glycogen to lactate and the lactate transfer to neighboring cells, such as neurons [117–120], might maintain neuronal survival in the penumbra where the lactate oxidation can occur thanks to a decreased but sufficient oxygen concentration [103]. Moreover, the level of the astrocyte glial cell line-derived neurotrophic factor (GDNF), known to inhibit neuronal apoptosis after IS and to decrease infarct volume [121, 122], is significantly decreased in the SHRSP astrocyte culture, in comparison to WKY cells [123, 124], but these results have to be confirmed *in vivo* [56].

Despite many limitations, the studies cited above have the virtue to switch from a research primarily focused on the neuronal consequences of IS to the astrocyte reaction. Up to date, these studies have mostly focused on SHR rats and *in vitro* cell culture experiments. The next challenge will be to explore astrocyte reactivity in other hypertension models and other animal strains. Secondly, new tools and techniques are needed to explore astrocytes with a higher sensitivity since the most used astrocyte marker GFAP does not identify all astrocytes, nor is it sensitive enough to equally identify astrocyte phenotypes [125, 126]. In the past few years, new *in vivo* and *in vitro* techniques have been developed in this direction to specifically explore astrocyte biology (double or triple immunostaining, flow cytometry, or genetic models such as the *gfap-Cre* and *Aldh1l1-eGFP* lines [125]). These new techniques must now be applied to ischemic stroke models with concomitant hypertension to better understand the crosstalk between astrocytes, microglia, and neurons and to investigate if astrocytes could be a therapeutic target as suggested in a cell therapy study [127].

### 4.3. Inflammation after Ischemic Stroke Decreases Neurogenesis and Impairs Brain Plasticity

In the human brain, neurogenesis is induced after focal IS with newborn neurons (with double staining DCX and Ki-67) present in the peri-infarct area [128]. This was also demonstrated in different stroke animal models caused by middle cerebral artery occlusion, with a neurogenesis induction in the subventricular zone and dentate gyrus [127, 129–133]. Interestingly, the newly formed cells migrate to the peri-infarct area [134, 135]. Furthermore, neurogenesis after stroke is highly dependent on angiogenesis, with a reduced survival of neuroblasts when angiogenesis is inhibited [127]. Unfortunately, the literature is scarce regarding neuronal damages and neurogenesis response after IS with concomitant hypertension [136]. Once again, mostly assessed in SHR and WKY strains, the potential diminished neurogenesis in hypertension was first described in a global ischemic model with twenty minutes of bilateral carotid artery occlusion [137]. In this setting, SHR presented higher rates of neuronal death in the CA1 hippocampus region secondary to extracellular glutamate increase [137]. *In vitro*, cortical neuron vulnerability was assessed during H/R in culture conditions and SHRSP yielded a strong neuronal degeneration compared to WKY after 36-hour hypoxia [138], with similar results for hippocampal neurons [139]. Using the same model of global cerebral ischemia by bilateral carotid occlusion, Negishi et al. further tried to assess the mechanisms that led to neuronal death in hypertensive conditions. After 20 minutes of occlusion, SHRSP in comparison to age-matched WKY presented an increase in 2,3-dihydrobenzoic acid, a biomarker of hydroxyl radicals, in the hippocampus [140]. These studies suggest an increased neuronal susceptibility to ischemia in the hypertension setting, particularly in regions involved in neurogenesis. If chronic hypertension enhances neuroinflammation, thereby limiting neurogenesis [134] even without IS, it is highly probable that neuronal vulnerability will be increased after additional focal cerebral ischemia (Figure 1); however, such studies are still lacking. Indeed, nearly 80% of newly born neurons die within the first 2 weeks after IS [129], partly because of the proinflammatory environment that takes place after stroke [134, 141]. The aseptic inflammation occurring days after IS is reproduced by LPS injection. Microglial activation is probably a strong determinant, limiting neurogenesis [134, 142, 143] through proinflammatory cytokine release (IL-6, IFN-gamma, IL-1-beta, and TNF-alpha) [143–148]. As microglial activation is enhanced in hypertension models, microglial cells could have a major role in decreased neurogenesis.

## 5. Antihypertensive Medications Improve Functional Outcomes by Modulating Microglia and Astrocyte Activation

Finally, selecting antihypertensive therapies that have anti-inflammatory properties could enhance neurogenesis and improve clinical outcome. Cifuentes et al. recently reviewed the benefit of antihypertensive drug therapies in Alzheimer disease, highlighting different operating mechanisms according to the treatment used [149]. Applied to IS, angiotensin II AT1 receptor blockers (ATRB) have been shown to limit and control brain inflammation after diverse stimuli (mostly LPS) [150] and modulate microglial polarization in the hippocampus [151, 152] leading to a decreased rate of dementia. Interestingly, these results were also confirmed with another class of antihypertensive drug, the angiotensin-converting-enzyme (ACE) inhibitor, which also antagonizes angiotensin II effects [153]. Specifically, ATRB and ACE inhibitors are attractive drugs, widely prescribed, with tremendous immunomodulatory effects evaluated in different brain inflammation models [154]. As angiotensin II is one of the hallmarks of hypertension genesis, counteracting its effects by ATRB or ACE inhibitors after IS in order to polarize microglia and astrocytes towards an anti-inflammatory phenotype to favor neurogenesis needs to be further addressed. The fact that their therapeutic effects seem to be independent of their blood pressure-lowering properties underlies the complexity of hypertension pathophysiology and illustrates how, beyond hemodynamic features, this vascular risk factor creates a deleterious environment for the brain plasticity and impacts prognosis.

## 6. For the Future?

This review highlights the value of considering vascular risk factors such as hypertension in preclinical models of IS. From a translational point of view, this element is crucial and largely explains the failure of neuroprotective treatments in stroke when transferred from the bench to the bedside. Significant efforts remain to be done in order to diagnose hypertension as early as possible and to treat efficiently this chronic and silent disease in order to limit the parenchymal consequences described above. Apprehending the consequences of hypertension on its proinflammatory side seems relevant as it has become the case for atherosclerosis [155]. It is likely that in the near future, considering the presence of vascular risk factors, such as hypertension, and inflammatory biomarkers, the therapeutic management will be tailored to the patient's phenotype. As inflammation is enhanced after IS in animals with hypertension, upcoming preclinical studies should focus on the possible impact of immunomodulation therapies to be proposed to this specific category of patients who, being hypertensive, would exhibit a more pronounced inflammation and therefore a worse prognosis.

The immunological and inflammatory vision of BP management is interesting, particularly in the field of cognition. Despite 4 years of follow-up, the recent SPRINT MIND study did not find any benefit of an intensive BP treatment strategy (SBP< 120 mmHg) in the reduction of probable dementia [156]. However, this study displayed a trend towards a reduction in mild cognitive impairment. The recent SPRINT MIND substudy found a smaller increase in white matter lesions in the intensive group but a greater decrease in total brain volume in this group, although with small differences [157]. In the past few years, several studies have raised concerns about the possible negative impact of excessive BP reduction in elderly patients (>80 years old) [158]. A key reason for these concerns is that the current randomized controlled trials evaluating the impact of BP treatments on cognitive decline are mainly based on SBP, while the mean arterial pressure is the most important BP parameter for cerebral perfusion pressure assessment. Nicely written by Saper, SBP reductions applied to elderly and fragile patients, often suffering from isolated systolic hypertension and its consequences on cerebral autoregulation with a curve shifted to the right, but also arterial stiffness, could lead to a decreased cerebral perfusion [159]. This might not be the case for young hypertensive patients with robust arterial compliance. These data reinforce the relevance of capturing hypertension by its inflammatory side. At equal BP values, antihypertensive therapies would allow to modulate inflammation, without significantly decreasing BP values. This approach has not yet been evaluated in the field of cognition, and further studies are urgently needed to assess if the antihypertensive medications and their immunomodulatory properties could have an impact on dementia incidence, particularly after stroke.

## Figures and Tables

**Table 1:** Hypertension models in the rodent. BP: blood pressure; SBP: systolic blood pressure; DBP: diastolic blood pressure; HTN: hypertension.

Model type	Hypertension characteristics	Animals	Morbidity	Reproducibility	Feasibility	Cost
*Pharmacological models*						
Angiotensin II	Severe HTN BP increase within 6-10 days	Mice Rats	Moderate	High	Moderate Subcutaneous pump	Low
Deoxycorticosterone acetate (DOCA)	Severe HTN Usually in combination with unilateral nephrectomy and high-salt diet BP increase within 3 weeks	Mice Rats	Low	High	High	Low
N-Nitro-L-arginine methyl ester (L-NAME)	Moderate HTN: SBP = 164 ± 6 mmHg at 4-6 weeks (rats) Inhibition of NO synthase	Mice Rats	Low	High	High	Low

*Genetic models*						
Spontaneously hypertensive rats (SHR)	Severe HTN SBP > 180 mmHg by 6 months postnatal	Rats	Low	High	High	Low
Stroke-prone SHR (SHRSP)	Extremely severe HTN (>SHR)	Rats	High High incidence of stroke	High	High	Low
Dahl salt-sensitive rat	Severe HTN with high-salt diet (8% NaCl) Moderate HTN with low-salt diet (0.4% NaCl)	Rats	High Low	High	High	Low

*Surgical models*						
Two-kidney one-clip model (2K1C)	Secondary (renovascular) HTN with increased renin-angiotensin-aldosterone activation >20 mmHg higher for SBP/DBP than sham-operated controls 4 weeks after clipping [160] SBP > 150 mmHg 3 weeks postsurgery [161] SBP peak mean value: 172 ± 25 mmHg at 47 ± 12 days postsurgery [161]	Rats Mice	Moderate	Moderate (70%) [161] Influenced by clip type, diet, and animal age [26]	Rats: high Mice: medium	Low
Two-kidney two-clip model (2K2C)	Secondary (renovascular) HTN with increased renin-angiotensin-aldosterone activation SBP > 150 mmHg 3 weeks postsurgery SBP peak mean value: 215 ± 23 mmHg at 172 ± 48 days postsurgery [161]	Rats Mice	High Increased incidence of stroke (62%) [161]	High (100%) [161] Influenced by clip type, diet, and animal age [26]	Rats: high Mice: medium	Low
One-kidney one-clip model (1K1C)	Secondary (renovascular) HTN with increased renin-angiotensin-aldosterone activation >35 mmHg higher for SBP/DBP than sham-operated controls 4 weeks after clipping, more rapid than 2K1C [160] SBP > 170 mmHg 3 weeks postsurgery SBP peak mean value: 196 ± 18 mmHg at 47 ± 23 days postsurgery	Rats Mice	High Acute kidney failure (23.3%) [161] Increased incidence of stroke (23.3%)	High (76%) [161] Influenced by clip type, diet, and animal age [26]	Rats: high Mice: medium	Low

**Table 2:** Summary of focal stroke models in the rodent. MCAO: middle cerebral artery occlusion; MT: mechanical thrombectomy; ET-1: endothelin-1; IC: internal capsule.

Model type	Transient/permanent	Topography	Standard neurological scores	Morbidity	Reproducibility	Translational approach	References
Intraluminal suture MCAO model	Permanent Transient	Large infarct Cortical and subcortical (according to the duration of ischemia)	Highly impaired	High	Moderate	No craniectomy Conflicting data in the literature: model of malignant ischemic stroke versus regain of interest with MT for ischemia/reperfusion models	[34, 35, 38]
Distal branch electrocoagulation or ligation	Permanent	Small infarct Cortical	Subnormal	Low	High	Craniectomy with dura mater incision Risk of brain electrocoagulation induced lesions	[34, 35, 38]
Photothrombosis	Permanent	Small infarcts Cortical or subcortical infarcts	Subnormal	Very low	High	Far from reality: no penumbra Useful for lacunar strokes	[34, 35, 38]
Endothelin-1	Transient	Variable size Subcortical infarcts	Subnormal	Low	Moderate	Far from reality: minimal edema Useful for lacunar strokes	[34, 35, 38]
Stereotaxic malonate injection	Permanent	Focal small infarct Depending on topography (stereotaxic): lacunar or cortical	Impaired	Low	High	Useful for lacunar strokes Can specifically target IC Less morbidity than ET-1 model Far from reality for cortical strokes	[162]

## Abbreviations

Ag II: Angiotensin II
ATRB: Angiotensin II receptor blocker
BP: Blood pressure
CEI: Conversion enzyme inhibitor
CNS: Central nervous system
DCX: Doublecortin
DOCA: Deoxycorticosterone acetate
DSS: Dahl salt sensitive
GFAP: Glial fibrillary acidic protein
IFN: Interferon
IL: Interleukin
IS: Ischemic stroke
L-NAME: N-Nitro-L-arginine methyl ester
LPS: Lipopolysaccharide
MCAO: Middle cerebral artery occlusion
NOS: NO synthase
SHR: Spontaneously hypertensive rat
SBP: Systolic blood pressure
SHRSP: Spontaneously hypertensive rat stroke prone
TNF: Tumor necrosis factor
VCI: Vascular cognitive impairment
WKY: Wistar Kyoto rat.
